# Which factors influence mobility change during COVID‐19 in Germany? Evidence from German county data

**DOI:** 10.1111/rsp3.12537

**Published:** 2022-05-11

**Authors:** Andree Ehlert, Jan Wedemeier

**Affiliations:** ^1^ Faculty of Business Studies Harz University of Applied Science Germany; ^2^ Research Area ‘Economics of cities and regions Hamburg Institute of International Economics (HWWI) Germany

**Keywords:** county data, COVID‐19 pandemic, mobility, regional interaction, spatial econometrics

## Abstract

This study analyzes the role of regional demographic, socioeconomic, and political factors in mobility changes during the COVID‐19 pandemic in Germany. Spatial econometric models are applied using data from the 401 counties in Germany. The model incorporates measures to reduce potential endogeneity effects. Our results show that mobility change shows significant socioeconomic heterogeneity, which could affect future policy measures to contain the pandemic. For example, case numbers and the share of academics are negatively associated with changes in mobility. On the contrary, a region's mean age and rural location have a positive impact. Political and economic implications of the results are discussed. The findings point to a possible reorganization of spatial, economic, and social activities beyond the course of the pandemic.

## 1 INTRODUCTION

The coronavirus disease 2019 (COVID‐19) pandemic has been one of the greatest social and economic challenges of recent years, with approximately 427 million infections and 5.9 million deaths worldwide (as of February 25, 2022). The COVID‐19 pandemic is still in full swing, although the momentum and lethality has slowed in some countries, in part because of vaccination success. Policymakers have responded with a variety of tools to limit mobility and, thus, contacts and chains of infection.

In Germany, measures to restrict contacts were implemented for the first time in March 2020. Examples include direct mobility restrictions, temporary entry restrictions to certain federal states or counties (*Kreise*), and (nighttime) curfews. Indirect mobility restrictions consist of repeated appeals to the population to avoid private and tourist travel, the closure of restaurants, cafés, and leisure facilities, and self‐motivation (caution and insight) to refrain from contacts and travel. From an economic point of view, therefore, a bundle of measures has increased the individual costs of mobility (transaction costs) and, at the same time, reduced its attractiveness (utility).

This paper joins a growing body of work examining the impact of the COVID‐19 pandemic on economic activity. For example, Ferreira et al. (2021) discuss worldwide interdependencies and supply chain disruptions based on different economic scenarios. They highlight how international trade linkages may be hit by pandemic shocks. Our analysis of mobility change elucidates the empirical impact of policy measures to contain COVID‐19 in light of population behavior adjustment varying regionally with respect to socioeconomic characteristics. The results have broad economic, social, and policy implications. While the question of the macroeconomic impact of mobility changes has been discussed in Deb et al. (2021), for example, there are few studies analyzing the relationship between social status and the associated ability to effectively restrict one's mobility (in terms of mobility restriction as a luxury good, see, e.g., Huang et al., 2021). Related to this is the political possibility of deriving, for example, regionally specific and thus more targeted measures than before, if it is known which and how socioeconomic factors limit or promote mobility change in the wake of COVID‐19.

It is precisely at this point that our study complements the existing literature, leading this study to ask three previously unanswered questions:

- The first research question is based on the heterogeneity of mobility change in terms of possible regional, socioeconomic, health‐related, and political influencing factors at the ecological level. Several specific research hypotheses are associated with this question. For example, we assume that there is empirical evidence that academics may respond with stronger mobility reduction due to more flexible working conditions. We also expect stronger mobility reduction for urban versus rural counties owing to the broader options for limiting one's mobility in cities.
- Another question that has not yet been satisfactorily answered empirically is whether direct (legal contact restrictions) or indirect (e.g., as a voluntary response to high caseloads) contact restrictions lead to greater mobility restriction.
- The third question concerns possible regional interactions of mobility change between neighboring regions (global spillover effects). This refers to a mechanism of spillover of mobility restrictions in one's own region to neighboring regions, such as through learning or deterrence effects. In the literature, this issue has been raised, for example, by Long and Ren (2022) in view of the COVID‐19 pandemic.

Note that the analysis of spatial interaction effects (question (iii)) is of central importance also from a statistical point of view, as such interactions can have biasing effects on the answer to the first two research questions. In addition, when making political decisions, it is important to know whether external effects of own parameters and measures must be considered.

This paper is structured as follows. A brief overview of the literature is given in Section 2. The data used and the statistical methodology are covered in Section 3. The results are given in Section 4. Section 5 then discusses these results and relates them to their economic and political implications.

## 2 COVID‐19 AND MOBILITY: A BRIEF REVIEW OF THE LITERATURE

There is now an extensive literature on the relationship between changes in population mobility behavior and COVID‐19 dynamics. Most of the studies refer to transportation and (cross‐national) long‐distance travel as outcomes. For example, Linka et al. (2021) investigate the dynamics between mobility and COVID‐19 operationalized by global air traffic and local mobility. Their study demonstrates different intensities of disease dynamics by using passenger air travel, cell phone data, and COVID‐19 cases. For ten European countries – among others, Austria, Belgium, Denmark, France, Germany, and the United Kingdom – they find a time lag between mobility and disease dynamics of around 14.6 days on average. Moreover, it is discussed how local mobility data can help to identify super‐spreading events.

Kapitsinis (2020) is an example of a European Union (EU)‐wide study at the regional level. Explanatory factors of COVID‐19 dynamics are regional (NUTS 2 and NUTS 1) air quality, demographic variables, global interconnectedness, urbanization, and trends in health expenditures. In this study, regions with a high mortality rate are characterized by high shares of old people (65+ years). Regions with a weak health system exhibit higher COVID‐19 mortality rates.

Cutrini and Salvati (2021) analyze the spatial patterns during the first wave of the COVID‐19 pandemic in Italy. They discuss the relevance of multiple factors to explain the forces beyond the spatial dynamics of the pandemic such as airline networks, urbanization, economic sectors, and firm size for North and South Italy. Further, weather conditions have been discussed as a factor underlying the spread of COVID‐19, such as by influencing people's behavior of staying outside or inside, (which, in turn, affects the COVID‐19 infection rate). Palialol et al. (2020) and Santos et al. (2021), to name a few, also address this question. They find that the exogenous variations of a weather variable reduce the COVID‐19 transmission rate by approximately 9%.

Credit (2020) brings up underlying racial and ethnic disparities with respect to COVID‐19 infection and testing in the US cities Chicago and New York. White‐majority neighborhoods are characterized by lower infection rates than other racial groups. The findings suggest that socioeconomic factors may help to explain these differences. Hatayama et al. (2020) discuss that working from home and avoidance of mobility may grow with the level of income. The authors point out that, for example in service occupations, more activities can be expected to be transferred to the home office. In the context of COVID‐19, Crowley and Doran (2020) highlight differences in remote working potential by occupation and sector. Some occupations do not allow for remote work, so mobility and social distancing will not increase in certain occupations. However, the spatial distribution of economic activities due to sectoral or industrial clustering has an influence on the course of the pandemic. Where home office was not possible, the results showed a higher impact. For example, the US shutdown policies resulted in substantial increases in unemployment insurance claims (the policies caused a 12.4% increase of unemployment insurance claims according to Kong and Prinz, 2020).

Yilmazkuday (2021) discusses the welfare loss of travel restrictions in the United States. The costs are measured by corresponding distances. The results show an estimated 11% loss of welfare of at its peak. On the basis of this result, the paper proposes that the legal and regulatory framework should be aligned with regard to future pandemics. Iacus et al. (2020) study data on global air passenger traffic addressing the impact of travel bans on the air transport sector and its general economic consequences.

A more specific strand of literature deals with the associations between (COVID‐19‐induced) changes in mobility behavior and socioeconomic characteristics. The focus of the present study is to complement the literature in this field. For example, on the basis of a global online survey (approximately 600 participants), Dingil and Esztergár‐Kiss (2021) use a multinomial logit model to estimate the impact of sociodemographic and travel characteristics on mobility behavior (before and after COVID‐19 awareness, first wave). They find that age, income, travel distance, and mode are important influencing factors. Similar results are found by Czech et al. (2021), who relate the Human Development Index to mobility changes for 124 countries. For a global country dataset, Mendolia et al. (2021) examine how changes in information about the spread of the COVID‐19 pandemic affected community mobility, depending on government policies implemented at the time. They find that human mobility is significantly responsive to information about the spread of the pandemic.

Borkowski et al. (2021) study the impact of the pandemic on daily mobility behavior. The analysis is based on a sample of 1,069 people from Poland during March and April 2020. For data analysis, they apply a generalized linear model. In contrast to many publications that focus on long‐distance or neighborhood behavior changes, the paper by Borkowski et al. (2021) covers behavioral adaptation in the short distance of daily life. Explanatory factors include homeschooling, quarantine, and level of education. The model confirms the assumption that household composition is crucial for short‐distance travel avoidance. Here, the fear of COVID‐19 infection was also included as a control variable showing a significantly negative impact on mobility. Further, the study finds no significant effect for age or gender on the change in mobility, but does for occupation or car availability. Unlike our case, however, the study is based on individual data. The study also contains a very comprehensive literature review (also with respect to the selection of covariates), which will not be duplicated here.

Using network mobility data for Ontario, Canada, Long and Ren (2022) study the association between three different mobility measures and four socioeconomic indicators during the first and second waves of COVID‐19. They find strong associations between mobility and the socioeconomic indicators. They also discuss how the relationships between mobility and other socioeconomic indicators vary over time. Liu et al. (2021) study the extent to which socioeconomic factors are related to the reduction in population mobility for both 358 Chinese cities and 121 countries worldwide. The analysis is based on mobile phone data and Google mobility reports from early 2020. They find that a higher socioeconomic index is significantly associated with a greater reduction in mobility at both city and country levels.

Schlosser et al. (2020) investigate the impact of the pandemic on the mobility in Germany using mobile phone data. The authors emphasize that long‐distance travel in particular declined sharply. Koenig and Dressler (2021) address a question similar to our study (albeit with different data for Germany). Their mixed‐methods analysis assesses mobility changes in a rural region (Altmarkkreis Salzwedel). Their study is based on quantitative household surveys (301 persons) and qualitative telephone interviews (15 persons) on perceived mobility changes. Socioeconomic variables such as age, employment status, and income are included. Among other findings, the study shows that reductions in car trips were significantly associated with household income. Anke et al. (2021) analyze mobility behavior for a survey‐based dataset for Germany (about 4,000 participants) and find that curfew measures have little effect on mobility changes. However, neither of the above analyses is based on econometric models.

In selecting the possible socioeconomic factors influencing mobility change for our econometric model (see Sections 3 and 4), we largely followed the above literature on the association of mobility change with COVID‐19 dynamics. A selection of literature sources and possible hypotheses regarding socioeconomic factors influencing mobility behavior are summarized in Table 1.

**TABLE 1:** Literature‐based selection of covariates for the mobility change model

Variable	Justification for a possible influence on mobility and consideration in the model	Selection of possible sources
Household income	Income has an impact on occupational and leisure mobility; hypothesis: A higher income increases the possibility of restricting one's mobility.	Koenig and Dressler (2021); Czech et al. (2021)
Nursing home employees	Inclusion in the model is based on discussions in the German daily press, as nursing staff was hypothesized to be closely associated with pandemic dynamics	
Share of employed academics	Hypothesis: It is easier for academics to limit professional mobility	Tokey (2021)
Industry share	See share of employed academics	Borkowski et al. (2021)
Service share	See share of employed academics	Borkowski et al. (2021)
Tourist beds	Specific to Germany, as there were major constraints for this sector	
Mean age	The direction of the effect seems unclear a priori, since reduced professional but increased medical mobility are opposed in old age	Anke et al. (2021)
Women	Hypothesis: Negative influence, since the employment rate is still lower or employment is higher in areas with greater flexibility with regard to working from home	Anke et al. (2021)
Heart failure	Hypothesis: Negative impact on mobility due to greater concern about infection	Borkowski et al. (2021)
COPD	See above	Borkowski et al. (2021)
Physicians	Direction of impact unclear, but better local health care makes it easier to reduce mobility	Liu et al. (2021)
Pharmacies	See above	Liu et al. (2021)
People in need of care	Possibly a positive influence as mobility in the form of visits and home care cannot be reduced	
Population density	Hypothesis: Negative influence on mobility, as mobility restrictions are easier in densely populated regions	Tokey (2021); Long and Ren (2022)
Car density	See above	Anke et al. (2021)
Commuter balance	Hypothesis: Positive influence on mobility change, since commuting to work cannot always be restricted	
Share of foreigners	Direction of impact unclear	
Rural (0/1)	Hypothesis: Positive impact on mobility; see population density	Anke et al. (2021)
Pupils	Hypothesis: Positive impact on mobility since mostly face‐to‐face teaching was in place	Borkowski et al. (2021)
Childcare	See above	
Car travel time central city (*Mittelzentrum*)	Hypothesis: Positive influence, since mobility for supply purposes can hardly be reduced	Borkowski et al. (2021)
Broadband supply	Hypothesis: Negative impact as home office opportunities increase	
Commute over 150 km	See commuter balance	Borkowski et al. (2021)
Diff. hours of sunshine	The weather has an influence on leisure activities and thus mobility	Palialol et al. (2020); Santos et al. (2021)
Diff. temperature	See above	
Contact restrictions in public space (0/1)	Hypothesis: Negative influence, as the contact restrictions directly reduce mobility	Tokey (2021); Anke et al. (2021)
Wholesale and retail restrictions (0/1)		
Restrictions in tourism sector (0/1)		
Curfews (0/1)		

## 3 DATA AND METHODOLOGY

### 3.1 The data

This is an ecological study based on aggregated data at the level of the 401 counties in Germany, whose populations range from about 34,000 (Zweibrücken) to about 3,664,000 (Berlin). 1 As an outcome variable, the study uses the change in general mobility behavior at the county level in January 2021 (average values are calculated separately for weekdays and weekends for the entire month from January 4 to exclude the influence of the New Year's weekend) compared with the same period of the previous year. To map mobility at the county level, anonymized mobile communications data from the network of the communications provider Telefónica (Germany‐wide market share ≥ 30%) are used, which are processed by Teralytics and made available by the Federal Statistical Office (2021). The data provide an overview of the number of mobile devices performing a certain movement. Movements are recorded when a mobile device changes the cell. The target region of a movement is reached when the mobile remains in a cell for at least 30 min. Possible distortions due to regionally varying market shares of Telefónica are compensated for by Teralytics using an algorithm that extrapolates geographically differentiated local market shares to the total German population. 2

**FIGURE 1:**
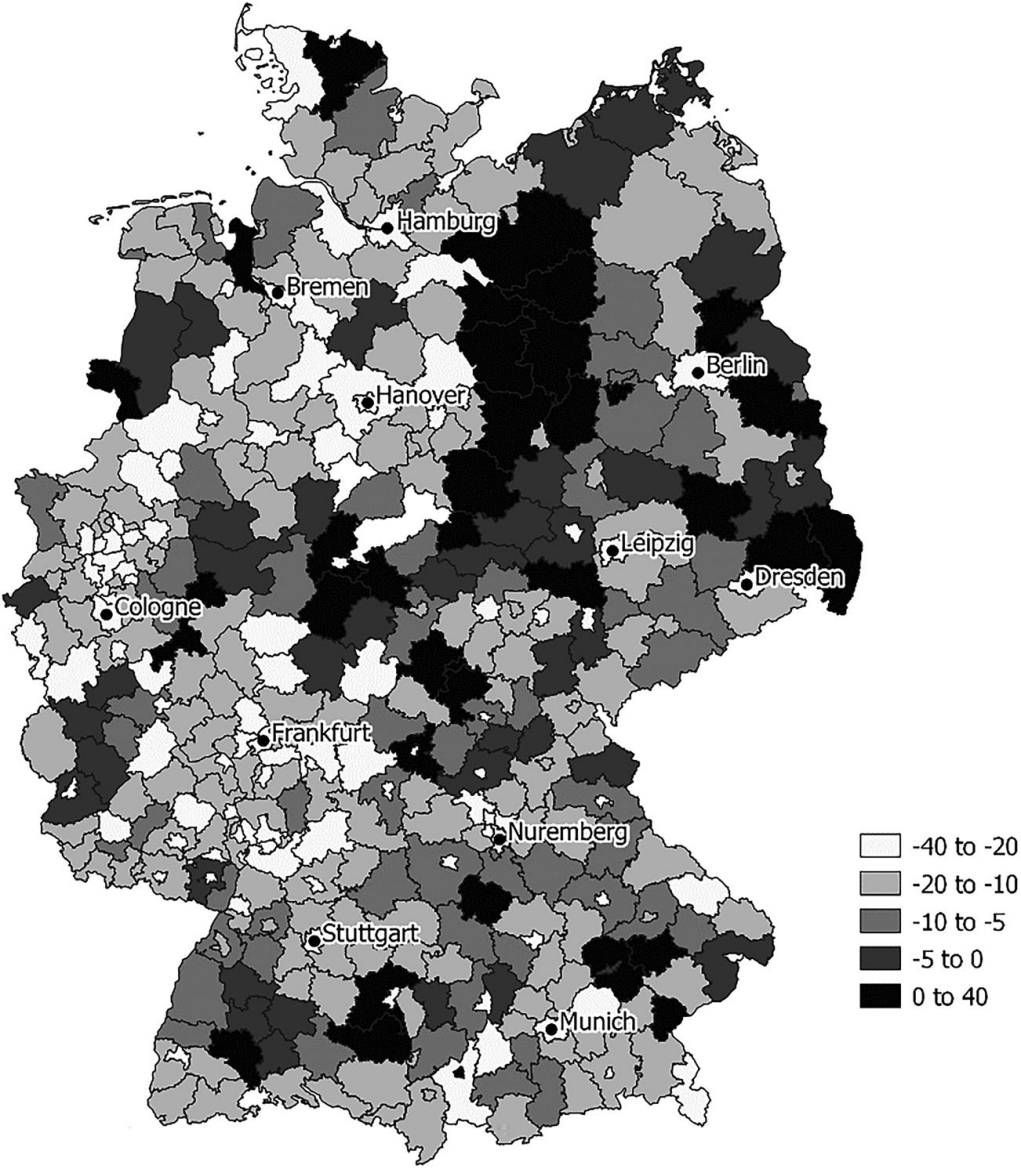
Mobility change on working days (January 2021 versus January 2020) at county level. Source: infas 360 (2021)

Empirically, a decline in mobility was observed in most of the regions (with few exceptions, mainly in the eastern part of Germany) (Figure 1). The month of January 2021 was chosen as the core period of the second COVID‐19 wave in Germany. The same month of the previous year, January 2020, was the month before the pandemic hit Germany.

In addition to the COVID‐19 case numbers, which can be considered an indirect (deterrent) factor in relation to mobility (see, e.g., Liu et al., 2021; Mendolia et al., 2021), a selection of covariates (measured also at the county level) was discussed in Section 2, where we largely followed the existing literature dealing with regional effects of COVID‐19. These factors may serve as possible explanatory variables for mobility change in our model (Section 3.2). An empirical overview of the dataset is provided in Table 2 (see the appendix for further details).

**TABLE 2:** Basic sample characteristics

Variable	Mean	Median	SD	*N*
Change in mobility in % (weekdays)	−13.413	−13.630	10.450	401
Change in mobility in % (weekends)	−13.274	−13.250	10.953	401
Cases January 2021 in surrounding counties (per 100,000 inhabitants)	590.146	520.230	212.982	401
Cases December 2020 in surrounding counties (per 100,000 inhabitants)	825.704	788.996	326.271	401
Cases first wave 2020 (per 100,000 inhabitants, own county)	212.222	172.171	163.145	401
Household income	1,872.561	1,869.000	215.765	401
Nursing home employees	97.709	96.800	23.279	401
Share of employed academics	11.958	10.300	5.170	401
Industry share	18.254	17.200	8.724	401
Service share	39.243	33.900	14.842	401
Tourist beds	41.776	27.000	49.309	401
Mean age	44.539	44.300	1.965	401
Women	50.597	50.600	0.645	401
Heart failure	3.845	3.530	1.420	401
COPD	6.455	6.400	1.503	401
Physicians	14.587	12.900	4.409	401
Pharmacies	27.004	26.100	4.900	401
People in need of care	428.125	424.200	106.029	401
Population density	533.748	198.000	702.713	401
Car density	579.160	593.000	70.980	401
Commuter balance	−10.362	−12.000	29.724	401
Share of foreigners	10.035	9.200	5.149	401
Rural (0/1)	0.339	0.000	0.474	401
Pupils	10.125	10.000	1.501	401
Childcare	32.269	28.800	12.077	401
Car travel time central city (*Mittelzentrum*)	6.786	8.000	5.548	401
Broadband supply	76.665	77.100	15.452	401
Commute over 150 km	4.412	4.000	1.354	401
Diff. hours of sunshine	−29.868	−31.970	17.547	401
Diff. temperature	−2.677	−2.650	0.605	401
Contact restrictions in public space (0/1)	0.910	1.000	0.286	401
Wholesale and retail restrictions (0/1)	1.000	1.000	0.000	401
Restrictions in tourism sector (0/1)	0.935	1.000	0.247	401
Curfews (0/1)	0.468	0.321	0.483	401

## 4 METHODOLOGY

Our econometric approach is a cross‐sectional ecological model based on data from the 401 German counties. The outcome variable used is the change in mobility between January 2020 and January 2021. In addition to the number of COVID‐19 cases, the factors influencing the change in mobility discussed in Sections 2 and 3.1 serve as covariates. It should be noted that there are currently no publicly available longitudinal data at the county level for the selected variables, and therefore the methods of time series analysis cannot be applied. 3

Three methodological challenges in the identification of influencing factors (at the ecological level) on mobility behavior in the course of COVID‐19 will be discussed in this section. These include, first, the problem of historical control, which results from the fact that all regions were hit simultaneously by the COVID‐19 shock. Second, the possible endogeneity of COVID‐19 case numbers in their influence on mobility change must be considered, and third, possible endogeneity due to the spatial feedback effects of mobility change.

Since this research is a historically controlled study, the first question of changed concomitant circumstances that would have varied on an annual basis even in the absence of the COVID‐19 pandemic and thus cannot be attributed to the pandemic must be carefully considered. Bias will be only partially avoidable from a statistical perspective but will be mitigated by the comprehensive covariates (variance in area, i.e., their possible influence on the changed accompanying and living circumstances). Conceivable biasing factors include general trends in regional mobility, holiday effects, and the influence of weather on mobility (e.g., for excursions). The last point is considered by the separate analysis of weekdays and weekends (cf. Table 3), as well as the inclusion of differences in sunshine duration and temperature between January 2021 and 2020 (whereby mobility in the month of January is certainly less influenced in this respect than in the summer months, so that some general robustness can be assumed for the study period). Furthermore, holiday effects are already considered in the data preparation by the Federal Statistical Office (2021). General trends in mobility over time are accounted for, at least indirectly, by its relation to socioeconomic variables and, of course, by the inclusion of a constant term (as a quasi‐linear time trend) as well as federal state dummies.

**TABLE 3:** Estimation results. Dummy variables for the federal states were included (results not shown)

	Weekdays			Weekends		
Coefficient estimates	Coef.	Std. err.	*p* > |*z*|	Coef.	Std. err.	*p* > |*z*|
Cases January 2021 in surrounding counties (per 100,000 inhabitants)	**−0.012**	**0.006**	**0.048**	−0.009	0.007	0.196
Cases December 2020 in surrounding counties (per 100,000 inhabitants)	0.000	0.004	0.946	−0.005	0.004	0.292
Cases first wave 2020 (per 100,000 inhabitants, own county)	0.000	0.003	0.896	−0.001	0.003	0.702
Household income	0.002	0.003	0.412	0.004	0.004	0.306
Nursing home employees	0.037	0.026	0.151	**0.053**	**0.030**	**0.077**
Share of employed academics	**−0.454**	**0.161**	**0.005**	0.032	0.188	0.863
Industry share	0.001	0.070	0.991	0.043	0.081	0.597
Service share	**−0.206**	**0.076**	**0.007**	**−0.243**	**0.088**	**0.006**
Tourist beds	**−0.016**	**0.010**	**0.097**	**−0.024**	**0.011**	**0.038**
Mean age	**1.268**	**0.508**	**0.013**	**1.494**	**0.594**	**0.012**
Women	**−1.745**	**0.855**	**0.041**	**−2.255**	**0.996**	**0.024**
Heart failure	0.111	0.483	0.818	0.256	0.570	0.654
COPD	**−0.739**	**0.407**	**0.069**	**−0.944**	**0.476**	**0.047**
Physicians	−0.075	0.234	0.749	0.194	0.273	0.477
Pharmacies	−0.198	0.123	0.108	**−0.281**	**0.143**	**0.050**
People in need of care	**0.017**	**0.008**	**0.029**	**0.027**	**0.009**	**0.003**
Population density	−0.002	0.001	0.127	−0.002	0.001	0.130
Car density	**−0.022**	**0.011**	**0.054**	**−0.030**	**0.013**	**0.022**
Commuter balance	0.032	0.032	0.321	0.003	0.038	0.927
Share of foreigners	0.250	0.183	0.172	0.077	0.215	0.719
Rural (0/1)	**2.830**	**1.106**	**0.010**	1.580	1.303	0.225
Pupils	−0.427	0.379	0.261	0.032	0.439	0.942
Childcare	0.141	0.095	0.138	0.097	0.110	0.380
Car travel time central city (*Mittelzentrum*)	**−0.219**	**0.126**	**0.082**	−0.145	0.147	0.323
Broadband supply	**−0.082**	**0.044**	**0.064**	**−0.116**	**0.051**	**0.024**
Commute over 150 km	−0.067	0.481	0.889	−0.036	0.560	0.949
Diff. hours of sunshine	−0.038	0.042	0.368	−0.030	0.050	0.554
Diff. temperature	1.119	1.449	0.440	−0.118	1.715	0.945
Contact restrictions in public space (0/1)	4.085	6.297	0.517	10.081	7.428	0.175
Wholesale and retail restrictions (0/1)	53.761	40.049	0.179	51.974	46.725	0.266
Restrictions in tourism sector (0/1)	1.710	6.345	0.788	8.749	7.444	0.240
Curfews (0/1)	−1.984	5.114	0.698	−9.368	5.987	0.118
Spatial lag	0.132	0.206	0.521	−0.167	0.262	0.523
Spatial autoregressive error	−0.039	0.576	0.946	0.147	0.591	0.804
LR chi‐squared (OLS)	0.430		0.809	0.420		0.813
Log likelihood	−1,337.321			−1,399.228		

The second challenge relates to the fact that case numbers cannot be considered an exogenous variable but are themselves very likely to be influenced by the dependent variable in feedback loops (classical econometric endogeneity problem). Plainly, it must be assumed that the change in mobility measured over a period of 1 year will have an effect on the number of cases in that period (this is, after all, the political rationale for inducing changes in mobility in the pandemic). The exact mechanism of action is of course unclear; refer to Gargoum and Gargoum (2021), and Krenz and Strulik (2021), who examine this influence. On the other hand, caseloads are a compelling part of the mobility change model, as they will in turn have an important indirect influence on mobility change through an information and deterrence effect.

Taking the above considerations into account, our identification strategy works as follows. We assume that the mobility change is driven, on the one hand, directly by contact and mobility restriction policies such as contact restrictions in public space, wholesale and retail restrictions, restrictions in the tourism sector, and curfews. Our corresponding variables (Table 2) reflect whether these restrictions were in effect on a given day in January 2021. On the other hand, we assume that mobility change is driven indirectly by the COVID‐19 case numbers (e.g., people adjust their lifestyle and mobility behavior in response to high COVID‐19 case numbers after being repeatedly encouraged to do so by policymakers) in addition to the above policy measures. See also Mendolia et al. (2021), who find that human mobility does respond in a significant way to information on the spread of the pandemic. We may thus assume that regional heterogeneity of case numbers serves as a central COVID‐19‐related parameter influencing mobility.

To reduce the endogeneity problem discussed above with the available cross‐sectional data (and to minimize possible distortions), we use an instrument instead of the case numbers of the respective county, namely the weighted average of the case numbers of the surrounding counties. Here, we choose as surrounding counties those that are at least 20 km and at most 150 km away from the respective (own) county. The reason for choosing the lower limit (20 km) is to reduce the presumed correlation with the error term as much as possible, since the influence of one's own mobility change on the number of cases in counties further away is greatly reduced. (This is still present through commuting linkages, but these are themselves included in the model as control variables.) At the same time, an upper distance limit must be found for a valid instrument, since an influence of case numbers by a deterrent effect should still plausibly exist. The exact limit is of course open to discussion, but for regions with a distance of more than 150 km it can be assumed that the deterrent effect of the case numbers on own mobility strongly decreases.

Third, in contrast to a standard linear model (OLS), our analysis takes into account the spatial distance of the observation units (counties) from each other. The reason for this is that the spillover effect caused by feedback effects of mobility from neighboring regions on one's own mobility (endogenous spatial interaction) should also be taken into account in the model, as otherwise a bias of the coefficient estimators may result (see, e.g., Elhorst (2014) for a discussion). Spatial statistical models (see below) reflect the fact that outcomes in one region may be influenced by outcomes and/or covariates in neighboring regions (spatial spillover effects) and/or a spatial autocorrelation of the residuals. This proposition can be explained, for example, via learning effects from neighboring regions or via spatial substitution of mobility. In the latter case, reduced mobility in one region is quasi‐substituted by increased mobility in neighboring regions. Spatial models can reduce potential bias of OLS estimation in the case of spatial spillovers and/or increase estimation efficiency. See, for example, Tokey (2021) for an application of such models with regard to COVID‐19 and mobility change.

To select concrete spatial econometric models, it is convenient to start with a model that is as general as possible (the so‐called general nesting spatial model (GNS) according to Elhorst, 2014), which includes the three sources of spatial lags discussed above:

$$\begin{array}{c} y=\mathrm{\rho Wy}+\mathrm{X\beta}+\mathrm{WX\theta}+u;\\ u=\mathrm{\lambda Wu}+\varepsilon . \end{array}$$

However, this general model is only weakly identifiable and is therefore rarely used in practice. It is therefore necessary to focus on a specific subclass of models. A common approach to model selection is to introduce at least one constraint of the form ρ=0,θ=0, or λ=0 and/or to base model selection on theory. Since we are particularly interested here in endogenous interactions (i.e., whether mobility change in neighboring regions exerts a spillover or learning effect on one's own region), we focus on the restriction θ=0, which corresponds to a spatial autoregressive confused (SAC) model and includes as special cases the widely used spatial autoregressive (SAR) and spatial error (SEM) models (see Elhorst (2014) for a discussion). SAR models are also proposed by Santos et al. (2021) in the context of COVID‐19, albeit the outcome variable there is incidence. Tokey (2021) uses an SEM model to analyze regional mobility data in the United States.

Technically, the spatial statistical models capture the neighborhood relationships using a so‐called spatial weighting matrix (i.e., a symmetric N×N matrix). This is based here on the geocodes (longitude and latitude of the circle centers) provided by the provider Opendatasoft (under the Creative Commons license). Specifically, the spmatrix command in Stata/MP 16.1 was used to create an inverse distance matrix from the coordinates, in which regions closer to each other are given a higher weight. The technical details of the spatial statistical models shall be omitted here with reference to the detailed discussion in Elhorst (2014).

## 5 RESULTS

Data analysis was performed using the spregress command in Stata/MP 16.1 (which effectively reduces to OLS when no spatial interactions are present). Table 3 presents the results for the SAC model, which we focus on here because it includes the spatial autoregressive and spatial error models (as well as OLS) as special cases. Before discussing the individual coefficient estimators, we briefly note that neither the likelihood ratio (LR) test (versus the OLS model, see at the end of the table) nor the estimated coefficients for spatial lag and spatial error turn out to be significant. Thus, the SAC model is not supported and the null hypothesis of a simple linear model cannot be rejected. However, we do not see this as a shortcoming of our modeling, but rather as an empirical result (unknown ex ante) with respect to a model, which allowed flexibility and thus a test with respect to spatial spillover effects, since these can be plausibly motivated from an economic point of view (cf. Section 3). Consequently, in Table 3 we report the results based on the spatial model, since it is the more flexible model and includes OLS as a special case. 4

To classify this result in terms of its actual meaning, the dynamics of possible spatial effects of mobility change should not be confused with the spillover effects of case numbers per se, which are more prominent in the public perception. This is because the latter are based on a quasi‐epidemiological process (‘viruses need mobility and proximity to spread’), whereas the dynamics of mobility are transmitted only indirectly (e.g., through mobility spillovers and learning and deterrence effects). In this context, note that the latter linkages are also controlled for directly in the model, such as by the commuter balance variable, so this may reduce the significance of the spillover effects according to the SAC model. This is also shown by the fact that, in a sensitivity analysis with a reduced model without covariates, significant spatial spillover effects do indeed occur. However, we do not consider this model without covariates to be appropriate in terms of its economic meaning and therefore refrain from discussing its results further.

The obtained coefficient estimators allow some interesting conclusions with respect to overall interpretation: Table 3 presents results for the change in mobility on weekdays (January 2021 versus January 2020) in the left‐hand three‐column block and results for weekends (in January 2021 versus January 2020, excluding the New Year's weekend) in the right‐hand block. In each case, the coefficient estimators, standard errors, and *p*‐values are given. Dummies for the 16 German states were included (not shown) in the estimation to account for the influences of state‐specific COVID‐19 measures.

Note that, as discussed in Section 3, the case numbers for December 2020 and January 2021 refer to the surrounding counties (20–150 km) to mitigate the endogeneity problem. This endogeneity problem does not exist for the case numbers of the first wave in 2020, so that the respective counties' own case numbers were used here. Death counts were not considered in addition to the case counts to avoid a multicollinearity problem (the death counts follow the case counts almost deterministically until January 2021 except for a constant factor).

Starting with the estimation results for weekdays, the significant negative association between the average (population‐standardized) case numbers in surrounding counties in January 2021 and the mobility change can be noted first, which is in line with the results discussed, for example, in Liu et al. (2021) for cities in China. A possible deterrent effect of high case numbers does not seem to last long, as the previous month's caseload shows no significant association with changes in mobility behavior. The share of employed academics and the share of service providers clearly show a significant negative association with changes in mobility. This also confirms results by Liu et al. (2021) and can be explained in particular by the higher home office rates in academic and service occupations.

The average age at the county level is significantly positively related to the mobility change, which may be explained, among other things, by the lack of influence of home office and homeschooling for older persons. Again, similar results are reported by Liu et al. (2021), where the proportion of people over 60 years shows a significant positive influence on intra‐city mobility change. Note, however, that age was found to be insignificant in the study by Borkowski et al. (2021) on individual survey data from Poland.

The regional share of women shows a significant negative association with mobility trends. One plausible reason may be the higher share of home offices in the service professions, the majority of which are held by women. Note, however, that gender is reported to be insignificant by Borkowski et al. (2021), but significant (at the 10% level) in a model of mobility change proposed by Dingil and Esztergár‐Kiss (2021).

Among the health variables, only chronic obstructive pulmonary disease (COPD) proportion seems to have a significant (negative) influence on the mobility change. Since people suffering from COPD are a high‐risk group in connection with a potential COVID‐19 infection, personal precautionary motives may serve as a plausible explanation. This result is also supported by Borkowski et al. (2021), where a variable termed ‘being afraid of infection’ is found to be significantly associated with a reduction in travel time during the pandemic.

In contrast, the number of persons in need of care has a significant positive coefficient. Since the data do not differentiate between institutional and home care, this result may simply reflect the lack of opportunity to substitute mobile outpatient care.

Car density exhibits a significantly negative coefficient. Although this may seem counterintuitive at first (see, e.g., Eisenmann et al., 2021), a high car density can also be seen as an indicator for a high potential of mobility reduction (e.g., use of cars for commuting). A similar reasoning applies to the significantly negative coefficient for car travel time to the nearest regional or urban center. An analogous result can be found in Borkowski et al. (2021), where a longer travel time before the pandemic is shown to be significantly associated with a larger reduction in mobility during the pandemic. In addition, the significant positive coefficient of rural regions is to be discussed. Here, the argument of a higher mobility requirement or higher costs of mobility avoidance for reasons of provision of general interest (shopping, commuting to work, medical care) applies. Results in the literature are mixed in this regard. Our result is supported by the analysis of Liu et al. (2021), while place of living is reported to be insignificant in Borkowski et al. (2021). As expected, higher broadband coverage, which is a prerequisite for reliable remote work, for example, will have a significant negative impact on the change in mobility.

The potential influence of temperature and sunshine duration on the change in mobility discussed in Sections 2 and 3 remains insignificant for both weekdays and weekends. However, it must be emphasized that these are monthly average values, whose distribution on weekdays or weekends was not differentiated.

Interestingly, direct political restrictions on mobility have no significant effect on the change in mobility. In this context, it should be noted that the regional heterogeneity of these restrictions (with the exception of curfews) is rather low in the period under review (January 2021) (Table 3). Also compare Anke et al. (2021), who also find only limited effectiveness of direct contact restrictions on mobility, and Liu et al. (2021), who instead report a significant reduction in mobility due to contact restriction (although this is a stricter variant of a lockdown than is captured with the four direct contact restriction variables in our dataset).

It is interesting to compare the estimated coefficients between weekdays and weekends. For example, in contrast to weekdays, the share of nursing home employees turns out significantly positive. This finding may be explained by the fact that a higher number of nursing home employees serves as a proxy for people living in nursing homes who, for example, get a visit from their relatives on the weekend.

Next, note that the negative effect of the share of academics on mobility (on weekdays) discussed above disappears when looking at weekends. This seems plausible with reference to high home office shares among academics, which play a minor role in leisure time on weekends. As on weekdays, the coefficient for car density is significantly negative on weekends. This could support the hypothesis that people refrain from leisure trips by car on weekends in the course of COVID‐19. The loss of significance of the rural location with regard to mobility at weekends also seems very plausible, since commuting to work and shopping are no longer an argument.

## 6 DISCUSSION AND CONCLUSION

The objective of the present ecological study is the empirical identification of associations between small‐scale mobility change and socioeconomic, demographic, health‐related, and political factors. We deliberately chose not to focus on the influence of mobility changes on the incidence of infections, which is the subject of many current epidemiological studies. Rather, we are concerned with the identification of ecological factors influencing mobility change in the course of COVID‐19 per se. (We again point out, as already discussed in detail in Section 3.2, that, for the case number variables, the direction of effect may be affected by endogeneity problems.)

Why is this question economically and politically relevant? First of all, it is important to understand people's empirical mobility patterns and their influencing factors in order to support policymaking in the phasing‐out of current measures or in future pandemic phases. Especially at the beginning of the pandemic, little was known about these relationships and policy measures had to be taken ad hoc without empirical guidance. The practical relevance of the question is also shown by the fact that the county regions in Germany react quite heterogeneously with respect to the change in mobility in the first year of the pandemic (Figure 1). This result alone would not be surprising, but we point out with our analysis that this heterogeneity is not randomly distributed but is in fact associated with the (measurable and known) socioeconomic heterogeneity of the regions. In the knowledge of these associations lies the great political opportunity to derive region‐specific, targeted measures for mobility reduction. For example, a maximum allowed number of face‐to‐face contacts (which applied as a measure in Germany in several pandemic phases) could also be made dependent on the socioeconomic profile of a region. Certainly, such considerations of heterogeneous measures will be politically difficult to implement and enforce. But one could apply knowledge of the links between the regional profile and its influence on mobility to, for example, resource management of the extent of control of measures in place.

According to the current state of the literature, our study is the first to answer this question using econometric methods based on regional data for Germany. In our model, we placed great emphasis on taking into account possible regional spillover effects of mobility change, which corresponds to our research question (iii) from Section 1 and was implemented using an SAC approach (Section 3.2). A main reason for choosing this model was that COVID‐19 has taught us that many behavioral changes work via regionally spreading information and the associated caution in the behavior of the population. In particular, it is reasonable to assume that the population in the pandemic learned to pay attention to the regional spread of infection numbers. It is also plausible that this cautionary mechanism (‘I imitate my neighbor's behavior’) plays a role in mobility patterns. Ultimately, we found no statistically significant spillover effects in our model in this regard. We emphasized in Section 4 that this does not mean that the model selection was wrong. Model selection should always be theory‐driven, since outcomes such as the nonsignificance of certain parameters cannot be known a priori. Moreover, the choice of possible spatial econometric model variants is very broad (see, e.g., Elhorst, 2014), and in an empirical test of these models against a simple OLS approach, only an a priori selection can be considered.

Following our three initial questions in Section 1, perhaps the most important result is that heterogeneity in mobility change can indeed be differentiated by statistically significant influencing factors (see our question (i); see also Czech et al., 2021, for an international perspective on this question). In addition to the caseload (which is also reported in the literature such as in Liu et al., 2021), we find some results in line with expectations (cf., e.g., the discussion on the share of academics, the service share, or broadband coverage in Section 4). This empirically supports the presumption discussed in the literature and daily press about the urban exodus to the rural home office and, of course, higher home office rates in service occupations, which often require a higher level of education (Crowley & Doran, 2020; Kapitsinis, 2020).

However, we also see some nonobvious results such as for COPD share (where COPD can be considered an immediate risk factor for a severe or fatal COVID‐19 course). This can be compared, for example, with the result in Borkowski et al. (2021) on the variable ‘being afraid of coronavirus’, which is statistically significant in the same direction as our COPD variable for mobility reduction. Furthermore, the positive significant influence of the variable ‘people in need of care’ may also be regarded as a nonobvious result. In the German daily press (but less in the scientific literature), there was a narrative in this regard during the initial phase of the pandemic that inpatient facilities for elderly care acted as infection drivers. With regard to our question of the influence of elderly care on mobility, it can be discussed (with caution regarding the interpretation of the results of ecological studies) whether a high proportion of elderly care at the regional level may make it more difficult to reduce mobility (which may also be due to the fact that approximately 80% of those in need of care in Germany are cared for at home by mobile care professionals; cf., e.g., https://www.bmwi.de/Redaktion/DE/Artikel/Branchenfokus/Wirtschaft/branchenfokus-pflegewirtschaft.html).

Another rather unexpected result was that income showed no significant association with mobility change in our analysis at the regional level. This is in contrast, for example, to the discussion in Dingil and Esztergár‐Kiss (2021) or Liu et al. (2021), where a significant positive association between income and mobility reduction is reported (and justified by increasing opportunities for mobility reduction with increasing income). However, our study includes more and different covariates that may explain mobility reduction better than income (e.g., the share of academics), and therefore income itself may become an insignificant variable. Moreover (and this consideration applies to ecological studies in general), the transfer of results obtained at the regional level to the individual level is not necessarily given; see, for example, the discussion and examples in Elford and Ben‐Shlomo (2004) on this issue.

Also considered unexpected might be the significant negative impact of regional female share on mobility change. However, existing studies on previous epidemics also suggest that women might tend to be more accepting of prevention measures (Agüero et al., 2011). In addition, existing structural differences in the professional and personal roles of men and women could be discussed to explain this result.

At first glance, the answer to our research question (ii) is also unexpected, namely that direct contact restrictions had no significant impact on mobility events for our dataset. Since, as mentioned, we did not have a time‐series dataset available, that is, we could not identify whether the measures had a temporary effect, if any, within our 1‐year period, this result must be interpreted with caution. This includes, again, possible (distorting) endogeneity effects, since not only did the direct contact restrictions have an impact on mobility, but mobility itself also had an impact on the contact restrictions (which came about through a political decision‐making process).

Of course, such a discussion also raises the question: What remains of regional mobility change in the medium and long term after the COVID‐19 era? Ultimately, the answer is speculation, but some scenarios from the literature shall nevertheless be briefly mirrored with our results. Unsurprisingly, regions with a high share of academics and a high share of services can react more flexibly to mobility change (see above and, e.g., Borkowski et al., 2021). This could have consequences for the near future development of mobility, especially for commuting to work. Initial studies from the UK and the United States are now beginning to discuss how COVID‐19 might affect economic geography. It may appear that the pandemic crisis will stabilize regional economic divergence and bring an end to booming cities and disconnected places (Farmer & Zanetti, 2021; Hendrickson & Muro, 2020).

Will previously disadvantaged rural regions seize the opportunity and improve living and working conditions for academics? The impact of broadband (i.e., home office opportunities for high‐skill occupations) highlighted this in our findings. Farmer and Zanetti (2021) also predict that remote work will be performance‐linked on broadband connections and localized digital infrastructure. Empirical studies of the impact of exogenous shocks on mobility behavior and public transportation suggest that the COVID‐19 crisis could permanently alter social behavior (Gutiérrez et al., 2020; Wang, 2014).

With respect to health care, note that there is already pronounced regional heterogeneity, with (sick and elderly) care increasing local and regional mobility. Politically imposed nonmobility, as was the case at the beginning of the pandemic, may have social consequences such as a rejection of political action. Finally, the effects of age and gender on mobility need further discussion. For example, why do women respond to COVID‐19 with a higher mobility restriction? It can be discussed that women have again become more involved in homeschooling and childcare (see also Borkowski et al., 2021). Emancipation is not yet fully consolidated in this respect, and it remains to be seen how sustainable this development of partnership role models will be.

Among the limitations of our results is certainly the absence of continuous time‐series data for the study period with respect to small‐scale mobility changes. Thus, by comparing two points in time (January 2020 versus January 2021), we cannot identify when exactly the mobility change occurred. Looking at the pandemic trajectory, there are two plausible scenarios: either the mobility reduction was implemented at the very beginning of the pandemic, during a period of great uncertainty and caution among the population, or with the surge of the so‐called second wave at the end of our observation period in winter 2020/2021. Also, with the available data, we cannot determine whether the first wave brought a significant mobility reduction, which then dropped off again and did not build up again in the context of the second wave (e.g., due to habituation effects of the population). In this respect, our results refer only to the comparison of mobility immediately before the pandemic (January 2020) with the values at the peak of the second wave (January 2021).

Another limitation remains, of course, the issues with our identification strategy (already discussed in Section 3.2) in the context of a historical control. Was COVID‐19 actually responsible for the mobility change, or was it due to other exogenous factors? For the sake of perspective, it should be noted that this problem applies to all studies of COVID‐19, and we see at least sufficient cross‐sectional heterogeneity in the data and covariates across the regional data for the 401 counties.

Finally, the general limitations of ecological studies remain to be mentioned, especially in terms of the transferability of results to individual data. This should always be taken into account when interpreting our results. We are dealing with results at the aggregate level, which should only be used as such and, strictly speaking, can only provide hypotheses for conclusions at the individual level. In addition to the data protection problems associated with the analysis of individual data on mobility and socioeconomic covariates, it should be noted, however, that our envisaged policy conclusions are also measures at the aggregate level and not at the individual level.

## Footnotes

^1^
The German counties (*Kreise*) correspond to the NUTS‐3 regions in the official Nomenclature of Territorial Units for Statistics.

^2^
See https://www.destatis.de/DE/Service/EXDAT/Datensaetze/mobilitaetsindikatoren-mobilfunkdaten.html for details. The data are further processed by the Federal Statistical Office as part of an ongoing research project (e.g., with regard to regional granularity and possible distortions due to public holidays) and made publicly available. This study is based on the currently publicly available data.

^3^
The currently available data status of the covariates is presented in Table 1 (the definitions and reference years in Table A1). Time series data are not available for the target variable ‘mobility change’ at the present time; see https://www.destatis.de/DE/Service/EXDAT/Datensaetze/mobilitaetsindikatoren-mobilfunkdaten.html#methodische%20Herausforderungen.

^4^
Moreover, this test result is to be classified as ex post and is well known not to imply the ‘acceptance’ of the null hypothesis that an OLS model is the true model from a statistical point of view.

## APPENDIX A

**TABLE A1:** Variable definitions

Variable	Year	Definition	Source
Change in mobility in %	2021	Daily percentage change in mobility at the county level (January 2021 as compared with January 2020)	Federal Statistical Office
Cases January 2021 in surrounding counties (per 100,000 inhabitants)	2021	Confirmed COVID‐19 infections per 100,000 inhabitants in January 2021 in surrounding counties (distance between 20 km and 150 km)	RKI
Cases December 2020 in surrounding counties (per 100,000 inhabitants)	2020	Confirmed COVID‐19 infections per 100,000 inhabitants in December 2020 in surrounding counties (distance between 20 km and 150 km)	RKI
Cases first wave 2020 (per 100,000 inhabitants)	2020	Confirmed COVID‐19 infections per 100,000 inhabitants from January 1, 2020, to May 15, 2020 (own county)	RKI
Household income	2017	Average disposable income of private households per inhabitant in EUR	BBSR
Nursing home employees	2017	Personnel in nursing homes per 10,000 inhabitants	BBSR
Share of employed academics	2017	Percentage share of employees subject to social security contributions with academic degree in all employees subject to social security contributions at place of residence	BBSR
Industry share	2017	Employees at place of work in industry (WZ 2008) per 100 inhabitants of working age	BBSR
Service share	2017	Employees at the place of work in the service sector (WZ 2008) per 100 inhabitants of working age	BBSR
Tourist beds	2017	Beds in tourist establishments per 1,000 inhabitants	BBSR
Mean age	2017	Arithmetic mean of the age of the entire population in years	BBSR
Women	2017	Percentage of women in the population	BBSR
Heart failure	2017	Number of patients with a confirmed heart failure diagnosis in at least two quarters among the total number of patients treated by statutory health insurance (SHI)‐accredited physicians in a calendar year	ZI
COPD	2017	COPD diagnosis prevalence: Number of confirmed COPD diagnoses among all SHI‐insured patients over 40 years of age who received services from SHI‐accredited physicians at least once in the calendar year	ZI
Physicians	2017	General practitioners (GPs) per 10,000 inhabitants	BBSR
Pharmacies	2017	Population‐weighted linear distance to the nearest pharmacy	BBSR
People in need of care	2017	Persons in need of care per 10,000 inhabitants	BBSR
Population density	2017	Inhabitants per km^2^ area	BBSR
Car density	2017	Cars per 1,000 inhabitants	BBSR
Commuter balance	2017	In‐commuters minus out‐commuters per 100 employees subject to social security contributions at place of work	BBSR
Share of foreigners	2017	Percentage of foreigners in the population	BBSR
Rural (0/1)	2017	Summarized settlement‐structural district types: Rural area in contrast to urban area	BBSR
Pupils	2017	Number of pupils per 100 inhabitants	BBSR
Childcare	2017	Proportion of children under 3 years of age in daycare facilities as a percentage of children in the corresponding age group	BBSR
Car travel time central city (*Mittelzentrum*)	2018	Car travel time to the nearest regional or urban center in minutes: The area‐weighted average value of car travel times to the nearest regional or urban center. The accessibility calculations for motorized private transport are based on route searches in a road network model. The determination of the car speeds on which the road types are based depends on the state of development as well as on the settlement structure and topographical conditions	BBSR
Commute over 150 km	2017	Commuters with a commute of 150 km and more per 100 employees at the place of residence, statistical basis: Commuter matrices of the Federal Employment Agency	BBSR
Diff. hours of sunshine	January 2021 to January 2020	Monthly sum of sunshine duration in hours	infas 360
Diff. temperature	January 2021 to January 2020	Monthly average of average daily air temperature at 2 m altitude, in °C	infas 360
Contact restrictions in public space (0/1)	January 2021	Contact/assembly restrictions on private individuals in public spaces according to ordinances at the district level	infas 360
Wholesale and retail restrictions (0/1)	January 2021	Current restrictions on wholesale and retail trade according to the ordinance at the district level	infas 360
Restrictions in tourism sector (0/1)	January 2021	Travel restrictions in force in the country according to the decree at the district level/travel restrictions in force abroad according to the decree at the district level	infas 360
Curfews (0/1)	January 2021	Applicable curfews according to the ordinance at the district level	infas 360
